# Depression, anxiety, and stress in medical students in Peru: a cross-sectional study

**DOI:** 10.3389/fpsyt.2023.1268872

**Published:** 2023-11-28

**Authors:** Danai Valladares-Garrido, Pedro P. Quiroga-Castañeda, Iván Berrios-Villegas, J. Pierre Zila-Velasque, Claudia Anchay-Zuloeta, Marisella Chumán-Sánchez, Víctor J. Vera-Ponce, César J. Pereira-Victorio, Virgilio E. Failoc-Rojas, Cristian Díaz-Vélez, Mario J. Valladares-Garrido

**Affiliations:** ^1^Escuela de Medicina, Universidad Cesar Vallejo, Trujillo, Peru; ^2^Oficina de Salud Ocupacional, Hospital de Apoyo II Santa Rosa, Piura, Peru; ^3^Facultad de Medicina, Universidad de San Martín de Porres, Chiclayo, Peru; ^4^Universidad Nacional Daniel Alcides Carrión, Facultad de Medicina Humana, Pasco, Peru; ^5^Red Latinoamericana de Medicina en La Altitud e Investigación (REDLAMAI), Pasco, Peru; ^6^Sociedad Científica de Estudiantes de Medicina Veritas (SCIEMVE), Chiclayo, Peru; ^7^Instituto de Investigación en Ciencias Biomédicas, Universidad Ricardo Palma, Lima, Peru; ^8^Universidad Tecnológica del Perú, Lima, Peru; ^9^School of Medicine, Universidad Continental, Lima, Peru; ^10^Unidad de Investigación para Generación y Síntesis de Evidencia en Salud, Universidad San Ignacio de Loyola, Lima, Peru; ^11^Escuela de Medicina, Universidad Privada Antenor Orrego, Trujillo, Peru; ^12^Red Peruana de Salud Colectiva, Lima, Peru; ^13^South American Center for Education and Research in Public Health, Universidad Norbert Wiener, Lima, Peru; ^14^Oficina de Epidemiología, Hospital Regional Lambayeque, Chiclayo, Peru

**Keywords:** depression, anxiety, stress, COVID-19, medical students, Peru

## Abstract

**Objective:**

To determine the prevalence and factors associated with depressive, anxious, and stress symptoms in medical students in Peru, during the second pandemic wave of COVID-19.

**Methods:**

We conducted an analytical cross-sectional study in 405 medical students from a university in northern Peru. The DASS-21 instrument was used to evaluate mental health outcomes (depression, anxiety, and stress), and to investigate their association with socio-educational characteristics.

**Results:**

We found a prevalence of depressive, anxious, and stress symptoms of 71.6% (95% CI: 66.94–75.95), 71.9% (95% CI: 67.2–76.2), and 62.7% (95% CI: 57.8–67.4); respectively. Students with eating behavior disorders had a higher prevalence of depressive symptoms (PR: 1.35), anxious symptoms (PR: 1.27), and stress symptoms (PR: 1.31). The prevalence of depressive symptoms (PR: 1.57), anxious symptoms (PR: 1.27), and stress symptoms (PR: 1.24) increased in students who did not report regular physical activity. In addition, having almost always academic exhaustion increased the prevalence of depressive symptoms (PR: 1.46), stress symptoms (PR: 1.72). On the contrary, the prevalence of depressive symptoms (PR: 0.79), anxious symptoms (PR: 0.73) and stress symptoms (PR: 0.82) decreased in male students. Students who reported sleeping 8 or more hours daily had a lower prevalence of stress symptoms (PR: 0.82).

**Conclusion:**

Symptoms of depression and anxiety occurred in 7 out of 10 students, and stress in 6 out of 10. Among the factors associated with the presence of anxiety, depression, and stress were eating behavior disorder and not regularly exercising. Periodic evaluations of mental symptomatology are required and counseling should be promoted in medical schools.

## Introduction

The pandemic caused by COVID-19 has had physical and mental repercussions in all regions of the world and every professions since students, health personnel, general population and military personnel (1–4). In Latin America and the Caribbean, a prevalence of 35 and 32% of depressive and anxious symptoms was reported (5); and in Peru, 30 and 40% of young people between 19 and 26 years of age showed symptoms of depression and anxiety in the context of COVID-19, respectively (6). In relation to medical students, in an Asian country it was found that 31.9% of students showed symptoms of depression, 32.9% of anxiety and 14.6% of stress (7). In Latin America, a prevalence of 49.8% of severe anxiety, 30.4% moderate depression and 17.8% severe stress was identified (8) and in Peru, prevalence of anxiety (28.5%), depression (24.3%) and stress (13.0%) was found during the first wave of the pandemic (9). And among the factors that increased these psychological disorders were the female gender; family, social and economic changes, high educational level and fear of going through a new pandemic, having comorbidities, low social support and low physical activity (2, 10–13).

However, there is not enough conclusive evidence about the persistence of mental health symptoms due to the pandemic and the factors associated with their presentation in medical students after the first year of the COVID-19 pandemic, characterized mainly by periods of confinement and restrictions globally. Additionally, studies that have described our research question present some methodological limitations, such as the small sample size (14), measurement bias since there are little explored variables and that are potentially associated with mental health outcomes (eating behavior disorder, insomnia, having burnout syndrome, physical activity) (15–19), they have a descriptive epidemiological design (20–24) and have not been carried out under rigorous biostatistical methods (20, 25, 26). Also, the Peruvian population it is different to other countries because the Peru it was one of the most affected by the pandemic with high numbers of deaths, more than 200,000, with more of two millions of contagions (27), being the country most affected in Latin America, fact that gave the students more time in their houses by a period almost 3 years, situations that increases the mental health problems (28).

While there is extensive documented evidence about the impact of COVID-19 on the mental health of medical students (29–32), little or nothing has been explored about the persistence of the impact of the pandemic when they have already returned to face-to-face classes at their universities worldwide (33–36) even more so in Latin American countries like Peru, severely hit by the pandemic. Medical students have constantly been exposed to stressful and anxious factors, particularly due to exposure to SARS-CoV-2 during clinical rotations upon returning to their face-to-face classes. Therefore, this research is favorable to reinforce the implementation of long-term preventive and mental support interventions in future doctors. The evaluation of mental health during this transition period from virtual education to face-to-face education through this research helps to solidly understand the challenges and changes caused by the pandemic in medical education.

Therefore, the main objective of this study was to identify the factors associated with depression, anxiety, and stress in a medical school in northern Peru.

## Materials and methods

### Study design

An analytical cross-sectional study was conducted during the 2021-II academic semester among medical students at the San Martin de Porres University (USMP), Chiclayo, Peru, in the context of the second pandemic wave of COVID-19.

### Population and sample

The study population consisted of 1,325 medical students who were studying between the first and seventh academic year of the 2021-II academic semester of the Human Medicine career at the USMP. Students who were enrolled in the 2021-II academic semester and who answered the questions of the dependent variables of the research (depression, anxiety, and stress) were included. Students who did not voluntarily agree to participate in the research were excluded. The sampling was non-probabilistic for convenience. A representative sample of medical students was estimated, using the Epi Info 7.0 program. An estimated proportion of 37.3% (26), a precision of 5%, a confidence level of 95, and 20% for incomplete surveys were used, with a formula for known population (*N* = 1,325). This gave us a sample size of 340 participants. However, we were able to capture a larger number of participants (*n* = 405).

### Instrument

Depression Anxiety and Stress Scales (DASS-21): an instrument that has 21 items, with four response options in Likert format (It has not happened to me = 0, It has happened a little = 1, It has happened quite a bit = 2 and It has happened a lot = 3). It contains questions about depressive, anxious, and stress symptoms presented in the last 2 weeks. It is considered a brief scale, easy to administer and answer, with its interpretation being straightforward. The total score ranges from 0 to 63 points (37). The DASS-21 questionnaire comprises three distinct dimensions that assess different aspects of mental health. Each subscale is assessed through a point sum ranging from 0 to 21. These dimensions are Depression, including items 3, 5, 10, 13, 16, 17, and 21; Anxiety, encompassing items 2, 4, 7, 9, 15, 19, and 20; and stress, covering items 1, 6, 8, 11, 12, 14, and 18. The cutoff points used were as follows: normal (0–4 points), mild (5–6 points), moderate (7–10 points), severe (11–13 points), and extremely severe (14 points or more). Subsequently, responses were dichotomized into: absence of symptoms (<5 points) and presence of symptoms (5 points or more). Regarding the reliability analyses, for the Depression scale, a sensitivity of 88.5% and specificity of 86.7% were identified. For the Anxiety scale, with a sensitivity of 87.5% and specificity of 83.4% and for the Stress scale, a sensitivity of 81.5% and specificity of 71.4% (38). With internal consistency for the depression scale (0.85), anxiety (0.72), and stress (0.79) (39), validated in the Latin American population.

SCOFF Questionnaire: an instrument that has been designed to identify symptoms related to eating disorders (such as anorexia nervosa and bulimia nervosa) in the last 3 months. It presents five questions in total, each of which is answered with a “yes” or “no” (40). Each affirmative answer is worth one point, while negative answers are worth zero points. Therefore, the answers are valued on a five-point Likert scale, with a total score ranging from 0 to 5. A score of 2 or more indicates an eating behavior disorder. It presents adequate a sensitivity of 81.9% and specificity of 93.5% (41, 42).

### General data

This section was made up of socio-educational questions: academic year, age, sex, weight and height report, to then estimate body mass index, frequent alcohol and tobacco consumption report, academic burnout report to the question “do your career’s academic activities have you emotionally exhausted?,” regular physical activity report to the question “do you exercise regularly?,” sleep quality measured with the question “how many hours do you sleep?,” with response option of less than 8 h daily and 8 h or more, and report of currently living at parents’ house, own house or apartment rental.

### Variables

The dependent variables were depression, anxiety, and stress, which were operationally defined, according to the explanation made about the use of the DASS-21 scale.

The independent variables were age in years, sex (female, male), year of studies (first to seventh), report of frequent alcohol consumption (no, yes), report of frequent tobacco consumption (no, yes), report of regular physical activity (no, yes), body mass index (normal, overweight, obesity), hours of sleep (<8 h daily, ≥8 h daily) and eating behavior disorder (no, yes).

### Study procedure

After obtaining the necessary authorizations at the university and the approval of the ethics committee, we created a survey in the REDCap data entry system. This questionnaire was shared through the official social network groups (Facebook, WhatsApp) of all the years of study of the participating university. The survey was self-administered and students took an average of 12 min to complete the required information. Subsequently, the data obtained were exported to a spreadsheet in Microsoft Excel for quality control and analysis.

### Statistical analysis

The statistical analysis was performed in the Stata v.17.0 program.

In the descriptive analysis, absolute and relative frequencies of categorical variables were shown. In numerical variables (age), the best measure of central tendency and dispersion was reported, after evaluating the assumption of normal distribution.

In the bivariate analysis, the factors associated with the dependent variables, that is, the mental health outcomes (depression, anxiety, and stress) were investigated. For the categorical independent variables, the chi-square test was useful, after evaluating the assumption of expected frequencies. In the numerical variable age, the Mann–Whitney U test was considered suitable, after evaluating the assumption of normal distribution. A significance level of 5% was used.

In the simple regression analysis, the factors associated with mental health outcomes (depression, anxiety, and stress) were identified through the construction of generalized linear models, Poisson family, log link function, and robust variance. The variables that were associated in the simple regression were included in the multiple regression model using a parsimonic model with forward selection (*p* < 0.05) to estimate prevalence ratios (PR) and 95% confidence intervals (IC95%). Collinearity between the variables of interest was evaluated.

### Ethical aspects

The confidentiality of the participating medical students was maintained, since the questionnaires were anonymous. Their desire to participate in the research was consulted through the filling out of informed consent, before accessing the questionnaires of interest. The research was approved by the Ethics Committee of the San Martin de Porres University.

## Results

### General characteristics of medical students

Of 405 medical students, we found that the majority were women (66.7%), were in their third year of study (22.5%), slept less than 8 h daily (78.0%), always or almost always feel emotionally drained from their academic activities (42.4%) and did not regularly perform physical activity (51.0%). 7.9 and 4.0% reported frequent consumption of alcohol and tobacco, respectively. The prevalence of depressive, anxious, and stress symptoms was 71.6% (95% CI: 66.94–75.95), 71.9% (95% CI: 67.2–76.2), and 62.7% (95% CI: 57.8–67.4; Table 1).

**Table 1:** Características de participantes (*n* = 405).

Characteristics	*N* (%)
Age (years)^*^	21 (19–23)
**Sex**	
Female	270 (66.7)
Male	135 (33.3)
**Academic year**	
1st year	60 (15.0)
2nd year	51 (12.8)
3rd year	90 (22.5)
4th year	60 (15.0)
5th year	39 (9.8)
6th year	38 (9.5)
7th year	62 (15.5)
**Currently parents**	
Live together	306 (76.5)
Single	94 (23.5)
**Currently living in**	
My parents’ house	348 (87.0)
Own house	23 (5.8)
Apartment rental	29 (7.3)
**Hours of sleep (daily)**	
<8 h	312 (78.0)
≥8 h	88 (22.0)
**Body mass index (BMI)**	
Normal	275 (69.3)
Overweight	108 (27.2)
Obesity	14 (3.5)
**Eating behavior disorder**	
No	241 (59.7)
Yes	163 (40.4)
**Frequent alcohol consumption**	
No	373 (92.1)
Yes	32 (7.9)
**Frequent tobacco consumption**	
No	389 (96.1)
Yes	16 (4.0)
**Academic burnout**	
Never/almost never	232 (57.6)
Almost always/always	171 (42.4)
**Regular physical activity**	
No	204 (51.0)
Yes	196 (49.0)
**Level of depressive symptoms**	
Normal	115 (28.4)
Mild	68 (16.8)
Moderate	111 (27.3)
Severe	50 (12.34)
Extremely severe	61 (15.1)
**Level of anxious symptoms**	
Normal	114 (28.2)
Mild	28 (7.0)
Moderate	110 (27.2)
Severe	47 (11.6)
Extremely severe	106 (26.2)
**Level of stress symptoms**	
Normal	151 (37.3)
Mild	67 (16.6)
Moderate	89 (22.0)
Severe	77 (19.0)
Extremely severe	21 (5.2)

^*^Median (25th–75th percentile).

#### Factors associated with mental health symptoms (depression, anxiety, and stress), in bivariate analysis

In the bivariate analysis, we found that female students had a higher frequency of depressive symptoms (62.6% vs. 39.3%; *p* < 0.001), anxious symptoms (73.7% vs. 47.4%), and stress symptoms (50.4% vs. 37.8%; *p* = 0.017); compared to male. Students who reported having emotional exhaustion almost always/always had a higher frequency of depressive symptoms (69.8% vs. 44.2%; *p* < 0.001), anxious symptoms (77.5% vs. 55.8%; *p* < 0.001), and stress symptoms (63.9% vs. 33.3%); compared to those who never/almost never had such exhaustion. Students who reported not exercising regularly had a higher frequency of depressive symptoms (67.2% vs. 42.3%; *p* < 0.001), anxious symptoms (77.5% vs. 52.0%; *p* < 0.001), and stress symptoms (54.9% vs. 37.2%; *p* < 0.001); compared to those who reported exercising regularly. Additionally, students with eating behavior disorders had a higher frequency of depressive symptoms (70.5% vs. 44.4%; *p* < 0.001), anxious symptoms (78.5% vs. 55.6%; *p* < 0.001), and stress symptoms (56.4% vs. 39.4%; *p* = 0.001; Table 2).

**Table 2:** Characteristics associated with mental health symptoms (depression, anxiety, and stress), in bivariate analysis.

Characteristics	Depressive symptoms		*p* ^*^	Anxious symptoms		*p* ^*^	Stress symptoms		*p* ^*^
	No (*n* = 183)	Yes (*n* = 222)		No (*n* = 142)	Yes (*n* = 263)		No (*n* = 218)	Yes (*n* = 187)	
	*n* (%)	*n* (%)		*n* (%)	*n* (%)		*n* (%)	*n* (%)	
Age (years)^**¶^	22 (19–25)	21 (19–23)	**<0.001**	22 (19–25)	21 (19–23)	**0.007**	22 (19–24)	21 (19–23)	0.192
Sex			**<0.001**			**<0.001**			**0.017**
Female	101 (37.4)	169 (62.6)		71 (26.3)	199(73.7)		134 (49.6)	136 (50.4)	
Male	82 (60.7)	53 (39.3)		71 (52.6)	64 (47.4)		84 (62.2)	51 (37.8)	
Currently parents			0.209			0.786			0.558
Live together	143 (46.7)	163 (53.3)		106 (34.6)	200 (65.4)		162 (53.)	144 (47.0)	
Single	37 (39.4)	57 (60.6)		34 (36.2)	60 (63.8)		53 (56.4)	41 (43.6)	
Currently living in			0.470			0.626			0.236
My parents’ house	153 (44.0)	195 (56.0)		121 (34.8)	227 (65.2)		182 (52.3)	166 (47.7)	
Own house	13 (56.5)	10 (43.5)		10 (43.5)	13 (56.5)		16 (69.5)	7 (30.4)	
Apartment rental	14 (48.3)	15 (51.7)		9 (31.0)	20 (69.0)		17 (58.6)	12 (41.4)	
Hours of sleep (daily)			0.734			0.649			0.168
<8 h	139 (44.6)	173 (55.4)		111 (35.6)	201 (64.4)		162 (51.9)	150 (48.1)	
≥8 h	41(46.6)	47 (53.4)		29 (33.0)	59 (67.0)		53 (60.2)	35 (39.8)	
Body mass index (BMI)			0.782			0.834			**0.021**
Normal	124 (45.1)	151 (54.9)		98 (35.6)	177 (64.4)		160 (58.2)	115 (41.8)	
Overweight	49 (45.4)	59 (54.6)		35 (32.4)	73 (67.6)		48 (44.4)	60 (55.6)	
Obesity	5 (35.7)	9 (64.3)		5 (35.7)	9 (64.3)		5 (35.7)	9 (64.3)	
Eating behavior disorder			**<0.001**			**<0.001**			**0.001**
No	134 (55.6)	107 (44.4)		107 (44.4)	134 (55.6)		146 (60.6)	95 (39.4)	
Yes	48 (29.5)	115 (70.5)		35 (21.4)	128 (78.5)		71 (43.6)	92 (56.4)	
Frequent alcohol consumption			0.568			0.492			0.411
No	167 (44.8)	206 (55.2)		129 (34.6)	244 (65.4)		203 (54.4)	170(45.6)	
Yes	16 (50.0)	16 (50.0)		13 (40.6)	19 (59.4)		15 (46.9)	17 (53.1)	
Frequent tobacco consumption			0.156			0.457			0.478
No	173 (44.5)	216 (55.5)		135 (34.7)	254 (65.3)		208 (53.5)	181 (46.5)	
Yes	10 (62.5)	6 (37.5)		7 (43.7)	9 (56.3)		10 (62.5)	6 (37.5)	
Academic burnout			**<0.001**			**<0.001**			**<0.001**
Never/almost never	129 (55.8)	102 (44.2)		102 (44.2)	129 (55.8)		154 (66.7)	77 (33.3)	
Almost always/always	51 (30.2)	118 (69.8)		38 (22.5)	131 (77.5)		61 (36.1)	108(63.9)	
Regular physical activity			**<0.001**			**<0.001**			**<0.001**
Yes	113 (57.7)	83 (423)		94 (48.0)	102 (52.0)		123 (62.8)	73 (37.2)	
No	67 (32.8)	137 (67.2)		46 (22.5)	158 (77.5)		92 (45.1)	112 (54.9)	

*p-value of categorical variables calculated with the Chi-square test. **p-value of categorical - numerical variables calculated with the U test (Mann–Whitney). ^¶^Median - interquartile range.Estimates of significantly associated variables are highlighted in bold.

#### Associated factors with mental health symptoms (depression, anxiety, and stress), in simple and multiple regression analysis

We found that the factors associated with a higher prevalence of depressive symptoms were reporting infrequent physical activity (PR: 1.37; 95% CI: 1.23–1.52) and presenting an eating disorder (PR: 1.35; 95% CI: 1.21–1.50). On the contrary, male students presented a 21% lower prevalence of depressive symptoms (PR: 0.79; 95% CI: 0.63–0.99).

Regarding anxious symptoms, we found that these increase in students who reported physical inactivity (PR: 1.27; 95% CI: 1.20–1.45) and presenting an eating disorder (PR: 1.27; 95% CI: 1.16–1.39). On the other hand, the prevalence of anxious symptoms reduced by 27% in male students (PR: 0.73; 95% CI: 0.55–0.98).

Finally, the factors positively associated with stress symptoms were having academic burnout always and almost always (PR: 1.72; 95% CI: 1.14–2.59), reporting physical inactivity (PR: 1.24; 95% CI: 1.07–1.44), and having an eating disorder (PR: 1.31; 95% CI: 1.14–1.49). On the contrary, students who reported sleeping 8 or more hours daily had a 16% lower prevalence of stress symptoms (PR: 0.84; 95% CI: 0.74–0.97), likewise, the prevalence of stress symptoms reduced by 18% in male students (PR: 0.82; 95% CI: 0.74–0.90; Table 3).

**Table 3:** Factors associated with mental health symptoms (depression, anxiety, and stress), in simple and multiple regression analysis.

Characteristics	Depressive symptoms						Anxious symptoms						Stress symptoms					
	Simple regression			Multiple regression			Simple regression			Multiple regression			Simple regression			Multiple regression		
	RP	IC 95%	*p* ^*^	RP	IC 95%	*p* ^*^	RP	IC 95%	*p* ^*^	RP	IC 95%	*p* ^*^	RP	IC 95%	*p* ^*^	RP	IC 95%	*p* ^*^
Age (years)**¶	0.93	0.90–0.96	**<0.001**	0.94	0.91–0.97	**<0.001**	0.97	0.94–1.00	0.110				0.97	0.93–1.00	0.093			
**Sex**																		
Female	Ref.			Ref.			Ref.			Ref.			Ref.			Ref.		
Male	0.61	0.51–0.73	**<0.001**	0.79	0.63–0.99	**0.044**	0.64	0.49–0.82	**0.001**	0.73	0.55–0.98	**0.036**	0.73	0.60–0.90	**0.003**	0.82	0.74–0.90	**<0.001**
**Currently parents**																		
Live together	Ref.						Ref.						Ref.					
Single	1.13	1.03–124	0.007				0.97	0.90–1.05	0.518				0.92	0.75–1.13	0.472			
**Currently living in**																		
My parents’ house	Ref.						Ref.						Ref.					
Own house	0.77	0.44–1.34	0.363				0.86	0.61–1.21	0.408				0.63	0.32–1.25	0.194			
Apartment rental	0.92	0.70–1.19	0.536				1.05	0.84–1.31	0.631				0.86	0.49–1.52	0.620			
**Hours of sleep (daily)**																		
<8 h	Ref.						Ref.						Ref.			Ref.		
≥8 h	0.97	0.77–1.21	0.808				1.04	0.85–1.28	0.665				0.82	0.66–1.03	0.097	0.84	0.74–0.97	**0.014**
**Body mass index (BMI)**																		
Normal	Ref.						Ref.						Ref.			Ref.		
Overweight	0.99	0.87–1.12	0.902				1.04	0.93–1.18	0.441				1.32	0.07–1.64	**0.010**	1.21	0.92–1.59	0.160
Obesity	1.16	0.75–1.8052	0.488				0.99	0.58–1.71	0.991				1.53	0.93–2.51	0.087	1.35	0.92–1.99	0.117
**Eating behavior disorder**																		
No	Ref.			Ref.			Ref.			Ref.			Ref.			Ref.		
Yes	1.60	1.35–1.91	**<0.001**	1.35	1.21–1.50	**<0.001**	1.41	1.27–1.56	**<0.001**	1.27	1.16–1.39	**<0.001**	1.44	1.23–1.69	**<0.001**	1.31	1.14–1.49	**<0.001**
**Frequent alcohol consumption**																		
No	Ref.						Ref.						Ref.					
Yes	0.93	0.62–1.40	0.753				0.90	0.59–1.36	0.634				1.21	0.89–1.65	0.202			
**Frequent tobacco consumption**																		
No	Ref.						Ref.						Ref.					
Yes	0.65	0.29–1.43	0.288				0.83	0.51–1.33	0.453				0.78	0.34–1.78	0.558			
**Academic burnout**																		
Never/almost never	Ref.			Ref.			Ref.			Ref.			Ref.			Ref.		
Almost always/always	1.58	1.26–1.98	**<0.001**	146	1.19–1.81	**<0.001**	1.39	1.08–1.78	**0.009**	1.26	0.97–1.62	0.073	1.91	1.30–2.80	**0.001**	1.72	1.14–2.59	**0.010**
**Regular physical activity**																		
Yes	Ref.			Ref.			Ref.			Ref.			Ref.			Ref.		
No	1.57	1.44–1.72	**<0.001**	1.37	1.23–1.52	**0.001**	1.48	1.29–1.69	**<0.001**	1.27	1.20–1.45	**<0.001**	1.47	1.22–1.77	**<0.001**	1.24	1.07–1.44	**0.004**

*p-values obtained with Generalized Linear Models (GLM), poisson family, log link function, robust variance, cluster by academic year.Estimates of significantly associated variables are highlighted in bold.

## Discussion

### Prevalence of mental health symptoms

We found that 7 out of 10 students showed symptoms of depression (71.7%), where 27.3, 12.3, and 15.3% presented moderate, severe, and extreme-severe depression, respectively. This is lower than reported in an American study conducted in university students during the COVID-19 pandemic, in which the prevalence of depression was 80.6% (26). However, it is higher than studies conducted in Chinese and Ethiopian university students during the pandemic context, where the prevalence was 23.3% (43) and 46.3% (29), respectively. Also, before the COVID-19 pandemic, prevalences of 29.7% (44) and 27.2% (45) were found in regions of Nepal among medical students.

This high frequency of depressive symptoms could be explained by a substantial burden of depressive symptoms in this student population, and its explanation is multifaceted, involving both factors related to the COVID-19 pandemic and factors inherent to the nature of medical education. Firstly, it is important to consider the context of the COVID-19 pandemic. Adapting to mandatory social distancing measures imposed to contain the virus’s spread has profoundly disrupted people’s lives worldwide. Medical students are no exception to this, and the restriction on social interaction, isolation from friends and loved ones, and the decrease in normal social activities may have significantly contributed to the increased depressive symptoms. Furthermore, the uncertainty and fear associated with the pandemic, as well as constant exposure to information about the health crisis, may have had a negative psychological impact on students. Another relevant aspect is the abrupt transition to virtual education, which has become the norm during the pandemic. Medical students faced unique challenges, as much of their learning relies on clinical experience and in-person interactions with patients and classmates. The shift to a virtual environment may have resulted in a sense of disconnection, loss of practical learning opportunities, and an increase in the feeling of isolation. In addition to pandemic-related factors, medical education itself can be stressful and demanding. Medical students often face an intense workload, tight schedules, high academic expectations, and constant pressure to excel. This academic and professional pressure can contribute to the onset and exacerbation of depressive symptoms. Lastly, while the COVID-19 pandemic has played a role in the increased depressive symptoms among medical students, it is essential to recognize that several additional factors are at play. The combination of academic pressure, emotional demands, financial difficulties, and other challenges inherent to medical education can contribute to the high prevalence of depression in this student population (46, 47).

We found that 7 out of 10 students showed anxious symptoms; where 16.2 and 26.5% suffered from moderate and extreme-severe anxiety, respectively. This is similar to what was reported during the first months of the pandemic in the United States by Lee et al. where 16.1% of medical students presented symptoms of moderate anxiety (48). It also coincides with a study conducted in Peru, where 28.5% of medical students had moderate levels of anxiety in the context of the first pandemic wave (9). However, it differs from the medical student population in China, of which only 15.8% suffered from anxiety disorder according to the study conducted by Xiao et al. in the context of the first wave (49).

Several factors may contribute to this elevated prevalence of anxious symptoms. Firstly, it is important to acknowledge the impact of the COVID-19 pandemic, which disrupted the traditional medical education system (48). The absence of face-to-face clinical practices and the abrupt shift to virtual learning likely had negative effects on the mental health of students (48). Clinical exposure and hands-on experience are integral components of medical education, allowing students to develop practical skills, interact with patients, and gain confidence in their abilities (48). The sudden interruption of these crucial learning experiences may have generated anxiety and uncertainty among students, especially given the essential nature of clinical training in medical education. Moreover, the extended duration of remote learning in Peru, with a return to face-to-face classes after 2 years, as indicated by the Ministry of Education (MINEDU) (50), adds another layer of complexity to the situation (48). Prolonged periods of virtual education can intensify feelings of isolation, hinder peer interactions, and contribute to a sense of detachment from the medical community. The anticipation of transitioning back to in-person classes may have also caused anxiety, as students may worry about readjusting to clinical settings and the potential challenges associated with this transition. Additionally, it is crucial to consider individual differences in coping mechanisms and resilience. Not all students respond to stressors and changes in the same way, and some may be more susceptible to anxiety symptoms than others. The competitive and demanding nature of medical education, coupled with the stressors brought about by the pandemic, may have pushed some students to the threshold of anxiety.

We found that 6 out of 10 students showed symptoms of stress (62.8%), where 21.9 and 19% presented moderate and severe stress, respectively. Similar to what was reported in university students (66.1%) from Ecuador during the first wave of the pandemic (51). This is consistent with what was described in Brazil, where 57.5% of university students presented stress during the COVID-19 pandemic (12). Also, in Chile during the pandemic, a prevalence of stress of 52.9% has been evidenced in university students (52).

This high frequency of stress symptoms can be attributed to a constellation of interconnected elements, both intrinsic and extrinsic to medical education. First and foremost, the demanding nature of medical training itself plays a significant role. Medical students are exposed to an intensive workload characterized by rigorous coursework, complex subject matter, and the expectation of exceptional academic performance. Additionally, they contend with tight schedules, the need to juggle multiple responsibilities, and the pressure to excel in a highly competitive environment. The pursuit of medical knowledge and the acquisition of clinical skills often demand long hours of study and practice, which can lead to exhaustion and heightened stress levels. The disruption caused by the COVID-19 pandemic further exacerbated the stress experienced by medical students. The abrupt transition to virtual education posed challenges such as reduced social interaction, limited hands-on learning opportunities, and the potential for technical issues during online classes. This shift in the educational paradigm forced students to adapt quickly, and the uncertainty surrounding the pandemic’s trajectory and impact contributed to heightened stress and anxiety. Furthermore, the fear of contracting COVID-19 added an extra layer of stress for medical students. As individuals aspiring to work in healthcare settings, they were acutely aware of the risks associated with the virus and may have experienced anxiety about their own health and safety, as well as the health of their loved ones. Financial concerns stemming from the pandemic also weighed heavily on students. Many faced economic hardships due to family job losses or reduced income, which could have increased their financial stress. The combination of academic pressures, virtual learning challenges, health-related anxieties, and financial burdens likely synergistically contributed to the observed high prevalence of stress symptoms (19, 26).

### Factors associated with depression symptoms

Male students had a lower prevalence of depression. This is consistent with what was described in a study conducted in Peru during the first wave of the pandemic, which revealed that being a woman was associated with depressive symptoms (OR = 1.34) (53). However, it contrasts with what was reported by a study conducted in medical students in China where no association was evidenced between depression and gender (54). This association could be explained because women, due to certain factors to which they are exposed, such as domestic violence and sexual abuse, are often more prone to develop mental health disorders (55, 56). This added to the fact that the female population often takes on more responsibilities at an early age and it has been shown that they present cognitive disorders and hyperactivity more frequently (55, 57). Unlike males, who, having behaviors of impulsivity and irritability, often camouflage depressive symptoms (58).

University students who reported not exercising had a 37% higher prevalence of depression. Our result is similar to reported in a study conducted in the university population, exercising is usually associated with less prevalence of depression (59), a result supported by different systematic reviews (60, 61). This association could be explained because having mental health symptomatology generates an imbalance in the monoaminergic systems, so its management focuses on correcting this imbalance, before this physical activity has shown that it increases the levels of noradrenaline, the levels of “free tryptophan fatty acids” which conditions the increase of serotonin (involved in depression) and finally decreases the stress response of the hypothalamus-pituitary–adrenal axis (involved because its hyperactivity with the consequent secretion of cortisol has been seen altered in depression) (62).

Having an eating disorder increased the prevalence of depression by 35%. This result is supported by what was found in the general population of Australia, which identified that having an EAD increased the risk of having a moderate and extreme level of depression in the pandemic context by 50% (63). Also, in Bolivian students it was evidenced that having an EAD increased the risk of depression by 66%, it should be noted that the study was conducted in a pre-pandemic context (64). Different from what was described by another study (65) that concludes a bidirectionality between the variables because depression predisposes the development of some eating disorder. This association could be explained because both pathologies share common biological mechanisms such as alterations in the mesocorticolimbic circuits, generating a decrease in the sensation of hunger; the dysfunction of the brain-microbiome relationship, through early fullness and the development of nausea and vomiting; and an endocrine-entero deregulation leading to starvation (66). Situations that lead to a multidisciplinary approach due to the different facets involved in the association.

### Factors associated with anxiety symptoms

Being male reduced the prevalence of anxious symptoms in medical students by 27%. This is consistent with what was described by Sheshtawy et al. in Egyptian medical students during the COVID-19 pandemic, who evidenced that belonging to the male sex presents a lower risk of developing symptoms of anxiety (67). However, it contrasts with what was reported by Yuan et al. in Chinese medical students (OR: 0.783) (30) in the context of the pandemic and by Simegn et al. in Ethiopian medical students (29). This association could be explained because each gender in particular has notable differences within their types of personalities. In the case of males, they tend to be more objective and usually seek immediate solutions to problems, this could make them less prone to suffer anxiety (68). Also, it has been reported that women tend to suffer from appetite disorders, hypochondria, and anxiety more frequently in contrast to males (56).

Having an eating disorder increased the prevalence of anxiety by 27%. This is consistent with what was described in a study conducted by Attouche et al. in Moroccan medical students during pre-pandemic, where having an eating disorder was associated with an increase in anxiety. Also, it has been identified that having an EAD increased the risk of anxiety by 54%, it should be noted that the study was conducted in a pre-pandemic context (64). However, a bidirectional relationship has been identified between the variables due to shared reward circuits (69). This association is explained by the influence of anxiety on the domains of eating behavior, such as cognitive restriction, referred to decision-making according to each food to consume due to its calorie-based composition, where anxiety in these people is elevated; also, it has been seen that anxiety predominates in the other domain such as Disinhibition because it generates impulsive consumption of food as in binges; and finally in hunger due to its neurobiological connections (70).

### Factors associated with stress symptoms

Being male reduced the prevalence of stress symptoms in medical students by 18%. This is consistent with what was described by Awadalla et al. in Saudi Arabian medical students during the COVID-19 pandemic, who evidenced that belonging to the female sex presents a higher risk of developing symptoms of stress (71). However, it contrasts with what was reported by Atta et al. in Egyptian medical students, who reported that prevalence of stress was higher in male (>85.0%) (72). This can be explained by the fact that male students are more self-confident and have more resilience strategies than female students, in addition to having more social support, which leads to developing less stress (73).

Reporting burnout syndrome increased the prevalence of stress. This is consistent with what was described by Yusoff et al. in second-year medical students during pre-pandemic context (74), also, it has been evidenced that burnout syndrome presented a variance for the development of stress in 19% (75). However, it contrasts with what was reported by a study that evidenced a negative correlation between personal fulfillment (3rd dimension of burnout syndrome) and the level of stress, concluding that the higher the stress, the lower the burnout syndrome (76). This association could be explained because medical students represent a special group because they often face many academic demands throughout their medical training (77), which in the context of the pandemic the high level of stress was evidenced in 1 out of every 10 students (11), in addition to dealing with the constant pressure to be successful in their career, the long hours of study, and the reduced amount of time for their social activities (78).

Not exercising was associated with a higher prevalence of stress symptoms. This is consistent with what was described by Leuchter et al. in American medical students in a pre-pandemic context, who reveals that the greater the intensity of exercise performed, the lower the levels of stress (79). Similarly, in Mexican medical students, a positive association was found between physical activity and a lower level of stress in a pre-pandemic context (80). We did not find studies that contradict this association. This association could be explained because there are potential mechanisms through which exercise could act on the body’s stress response, especially through the hypothalamus-pituitary–adrenal axis or the circulation of glucocorticoids (81, 82). In addition, physical exercise helps in the stimulation of processes relevant for the proper functioning of the brain, among them the stimulation of neurogenesis and angiogenesis, and the positive regulation of growth factors such as brain-derived neurotrophic factor (83). Therefore, thanks to the promotion of the functioning of those brain regions that play an important role in stress-related conditions, exercise could be a protective factor for developing stress symptoms.

Students who reported sleeping 8 h or more had a lower prevalence of stress. This is consistent with what was described in a study conducted in medical students in Saudi Arabia in a non-pandemic context, who found that the greater the amount of rest, the lower the prevalence of stress (84). Also, in American medical students in the context of the pandemic, an association was identified between the number of hours slept (7 h) and the lower level of stress (85). However, we found a study that evidenced bidirectionality between the variables (quality of sleep and stress) explaining that dealing with the COVID-19 infection, uncertainty about the pandemic, social confinement, and decreased physical activity make up a multifactorial origin that each of the variables share (86), variables that affect structures involved in the mechanisms of sleep regulation such as the hypothalamus-pituitary–adrenal axis and the immune system (87).

Having an eating disorder, evaluated with the SCOFF questionnaire, increased the prevalence of stress symptoms by 31%. This is consistent with what was described in a study conducted by Iyer S et al. (88, 89) in Indian medical students in a pre-pandemic context, who found that the eating disorder increased stress. Also, in the context of the pandemic in French students, a positive association was found between the variables of eating disorder and stress in French medical students (90). However, it contrasts with what was reported by a study in medical students conducted in Malaysia in a pre-pandemic context that did not evidence an association between the variables of eating disorder and stress (91), it should be noted that a different evaluation instrument was used (EAT-26). In our country, a prevalence of EAD has been evidenced in 2 out of every 10 medical students in the context of the COVID-19 pandemic (3). Eating disorders have a multifactorial origin, so this association could be explained because most medical students usually face the career during their youth years, where in addition to (92) being exposed to the academic load and the probable lack of time management (93), they must also face their transition to adult life and sociocultural factors such as social pressure to have a certain physical appearance (94–96). All of this makes them more prone to suffer from eating disorders.

#### Correlates between anxiety, depression and stress

The strongest correlates found in our population were that more than half of the population between 1 and 7 medical students presented at least one of the negative mental health symptoms. There was a direct correlation between being female, not exercising, presenting an eating disorder, presenting burnout syndrome with the development of these negative symptoms, as opposed to presenting an optimal sleep quality with a rest of more than 8 h, demonstrating a negative association with these negative symptoms. Although the pandemic as an unforeseen event developed the ideal context to develop these negative factors, discarding the biological sex and unlike the students who did not present these symptoms. It is well known that the natural relationship between these pathologies (anxiety, depression and stress) represent “normal” symptoms that are acquired in the course of medical school (97). We are convinced that reversing these negative factors and strengthening protective factors in students is not only aimed at preventing the development of these negative mental health symptoms associated with implementing a healthy lifestyle and an adequate social life that become distant upon entering medical school, but also to prevent the development of these negative mental health symptoms associated with implementing a healthy lifestyle and an adequate social life that become distant upon entering medical school (98).

#### Relevance of findings in public health

This study provides information on mental health in a phase of the COVID-19 pandemic little evidenced (third wave of pandemic). The role of mental health assessment in a particular population like medical students is vital to be able to observe their evolution and the measures of associated factors. This study provides information that helps to understand the evolution of mental health symptoms in a country that was very affected by the pandemic, compared to other countries in the region. We evidenced variables that have been little evaluated [being male, not doing any exercise, and rest time (8 h)], variables that have not shown association in other studies in the context of the pandemic and results that to our understanding would be the first reported in our region (Latin America). We highlight that despite the decrease in the number of infections, negative mental health symptoms still persist. Therefore, we believe that periodic evaluations of these symptoms, the implementation and execution of mental health programs, and continuous accompaniment in those who present these negative symptoms, would correspond to the public health measures to be introduced in every institution to avoid their increase and complications of their non-management.

### Limitations and strengths

In relation to the limitations of the study, first, the cross-sectional design of the study did not allow us to identify causal relationships between the study variables, but as a strength, in our study we used validated instruments on anxiety (99), depression (100) and stress (101). Also, another limitation found in the study we formulated structured questions for the evaluation of sleep quality, physical activity, substance use, and burnout syndrome. As a second limitation, we identified selection biases stemming from the lack of representativeness because we focused on a single university. Additionally, the use of snowball sampling limits the generalizability of the results and could potentially introduce biases such as misclassification bias and response bias, as students with better internet connectivity or higher motivation to participate might be overrepresented or underrepresented in the findings. However, it’s important to emphasize that these limitations do not invalidate the research results. But as a strength, we covered a broad sample of students from various academic years and from a region where research is flourishing and has been significantly impacted by the COVID-19 pandemic. Furthermore, we were able to engage a student population that faced accessibility challenges due to the virtual classes prompted by the COVID-19 pandemic (102). This proved beneficial for capturing diverse and partially representative information while maintaining flexibility and adaptability in data collection for the research. As a third limitation is the potential measurement bias because the questionnaires were self-administered and being an instrument that does not represent the Gold Standard of diagnosis of these mental health symptoms (103), in addition to not covering variables the post-traumatic stress disorder (PTSD) that gives us information about the chronicity of the symptoms, but as a strength we covered various variables that evidence important information in the context of the pandemic (EAD, amount of rest hours, physical activity, and gender). This research has measured variables that have been widely in the first year of the pandemic, however, it generates evidence in this new period of normality (3rd wave of the pandemic).

## Conclusion

We identified that symptoms of depression and anxiety were present in 7 out of 10 students, and stress in 6 out of 10. Among the factors associated with the presence of anxiety, depression, and stress were the eating disorder and not regularly exercising. Given this, we recommend periodic evaluations of these symptoms and the corresponding counseling in each institution.

## Data availability statement

The dataset generated and analyzed during the current study is not publicly available because the ethics committee has not provided permission/authorization to publicly share the data, but it is available from the corresponding author upon reasonable request.

## Ethics statement

The studies involving humans were approved by San Martin de Porres University. The studies were conducted in accordance with the local legislation and institutional requirements. The participants provided their written informed consent to participate in this study.

## Author contributions

DV-G: Conceptualization, Data curation, Formal analysis, Investigation, Methodology, Validation, Visualization, Writing – original draft, Writing – review & editing. PQ-C: Investigation, Methodology, Validation, Visualization, Writing – original draft. IB-V: Investigation, Methodology, Validation, Visualization, Writing – original draft, Writing – review & editing. JZ-V: Investigation, Methodology, Validation, Visualization, Writing – original draft, Writing – review & editing. CA-Z: Investigation, Methodology, Validation, Visualization, Writing – original draft, Writing – review & editing. MC-S: Investigation, Methodology, Validation, Visualization, Writing – original draft, Writing – review & editing. VV-P: Investigation, Methodology, Validation, Visualization, Writing – original draft, Writing – review & editing. CP-V: Funding acquisition, Methodology, Project administration, Resources, Software, Supervision, Validation, Visualization, Writing – original draft, Writing – review & editing. VF-R: Funding acquisition, Methodology, Resources, Software, Supervision, Validation, Visualization, Writing – original draft, Writing – review & editing. CD-V: Investigation, Project administration, Resources, Supervision, Validation, Visualization, Writing – original draft, Writing – review & editing. MV: Conceptualization, Data curation, Formal analysis, Investigation, Methodology, Project administration, Software, Supervision, Validation, Visualization, Writing – original draft, Writing – review & editing.
